# Performance of the Self‐Controlled Case Series With Active Comparators for Drug Safety Signal Detection Using the French Administrative Healthcare Database (SNDS)

**DOI:** 10.1002/pds.70224

**Published:** 2025-11-14

**Authors:** Astrid Coste, Angel Y. S. Wong, François Haguinet, Andrew Bate, Ian J. Douglas

**Affiliations:** ^1^ Department of Non‐Communicable Diseases Epidemiology LSHTM London UK; ^2^ GSK Wavre Belgium

**Keywords:** claims data, pharmacoepidemiology, pharmacovigilance, real‐world data, self‐controlled case series, signal detection

## Abstract

**Background:**

The self controlled case series (SCCS) is one of the most promising methods for drug safety signal detection using real world data (RWD), and incorporating active comparators could potentially improve its performance by addressing time‐varying confounding by indication. The ‘Système National des Données de Santé’ (SNDS) is a large nationwide administrative claims database, which has not been used extensively for drug safety signal detection. While comparable in size to other RWD sources, it is unclear to what extent the performance of SCCS correlates with that in other sources.

**Objectives:**

This study aims to evaluate the performance of the SCCS with and without active comparators for signal detection in the French administrative healthcare database SNDS.

**Methods:**

We applied the SCCS to macrolide and fluoroquinolone antibiotics, using amoxicillin as the active comparator. Amoxicillin was chosen as an active comparator with similar indications. In total, 7 drugs and 30 outcomes from all organ classes were selected. We developed a reference set of 104 positive controls and 58 negative controls, using a taxonomy framework to ensure the selected drug outcome pairs are theoretically well suited to the SCCS design. The observation period lasted 2 years, with a 30‐day risk window after each dispensing. Diagnostic performance was measured using sensitivity and specificity with respect to the product labels.

**Results:**

The sensitivity and specificity of the SCCS without active comparator were 0.89 and 0.43, respectively, when limited to pairs with satisfactory power. Specificity increased up to 0.91 with active comparators; however, sensitivity decreased to 0.52.

**Conclusions:**

The SNDS is a useful data source for signal detection, particularly for outcomes captured in hospitals. Using a carefully designed reference set of drug‐outcome pairs well suited to the study design, the SCCS achieved satisfactory performance for signal detection in this database. In this study, the use of active comparators improved overall performance at the expense of greatly reduced sensitivity.

**Summary**

- The self‐controlled case series (SCCS) is a promising method for drug safety signal detection using real‐world data but has not been implemented routinely because of heterogeneous performance reporting.
- We explored SCCS performance in French administrative claims data with and without active comparators using a tailored reference set based on macrolides and fluoroquinolones drug labels.
- The sensitivity and specificity of the SCCS without active comparator were 0.89 and 0.43 respectively, when limited to pairs with satisfactory power
- Using a reference set of drug‐outcome pairs well suited to the study design, the SCCS achieved satisfactory performance for signal detection in this database. In this study, the use of active comparators improved overall performance at the expense of greatly reduced sensitivity.

## 1 Introduction

Post‐marketing drug safety signal detection can benefit from the size and coverage of data capture of real‐world databases to complement spontaneous reporting systems in order to detect new adverse drug reactions (ADRs). The Systeme National des Donnees de Sante (SNDS) is a powerful source of information for post‐marketing surveillance of drugs as it contains healthcare claims for the entire French population (approximately 68 million people). It was created in 2016 as a linkage of several existing databases, including claims from the public health insurance system and data from hospitals [1]. Although it has been used in several pharmacoepidemiologic studies [2], it has rarely been used for signal detection [3, 4]. The SNDS benefits from the large sample size of claims databases and lifelong follow‐up as often found in electronic health records (EHRs).

To determine the value of RWD for signal detection, there is a need to evaluate the performance of epidemiological methods at finding alerts in a hypothesis‐free context that is, irrespective of whether there is a suspected association. In practice, this is limited by the requirements of study designs, which can better accommodate certain types of drugs and outcomes. Previous RWD signal detection initiatives exploring method performance did not consider that epidemiological methods are differentially valid depending on the nature of the drug and outcome [5]. These projects focused on US claims and European EHRs but did not include the SNDS [6, 7], so it is unclear if their findings would replicate in this database.

Notably, the self‐controlled case series (SCCS) was reported to be one of the highest performing methods for signal detection [8, 9, 10, 11, 12, 13, 14]. Based on the SNDS data, two studies from a project named ALCAPONE reported Area under the receiver operating characteristic curves (AUCs) ranging from 0.64 to 0.94 for the SCCS depending on the outcome and analytical configuration. Each study investigated a single outcome, respectively acute liver injury and upper gastrointestinal (GI) bleeding, with a wide range of drugs [3, 4].

One of the advantages of self‐controlled designs is that they inherently adjust for time‐independent confounders. However, they are subject to confounding by indication since this type of confounding is time‐dependent [15]. Active comparator designs could handle time‐varying confounding by incorporating comparator drugs with a similar indication as the exposure of interest, but not expected to cause the outcome under study. Active comparators have been recently implemented in Self‐Controlled designs in hypothesis testing studies using Danish registry data [15], as well as in Hong‐Kong and British EHRs databases [16, 17], but not in the SNDS. Active comparators with SCCS have not been evaluated in signal detection, so their impact on the method's performance remains unknown.

The aim of this study is to evaluate the performance of the Self‐Controlled Case Series with and without active comparators for signal detection in the SNDS. We used a reference set specifically developed for this study with a taxonomy framework to ensure the selected drug outcome pairs are well suited to the SCCS design. Using a drug‐based approach, antibiotics were chosen for this study as they are prescribed in short courses in France, which is a requirement of SCCS. Antibiotics have a well‐established safety profile since they have been on the French market for several decades, which is important for performance assessment.

## 2 Methods

### 2.1 Data Source

The ‘Système National des Données de Santé’ (SNDS) is a nationwide administrative claims database, covering around 99% of the French population (68 million people) [18, 19]. It was created in 2016 as a grouping of existing databases [1]. The SNDS contains pseudonymised data, including outpatient data (date of reimbursed drug dispensation and number of units, status of long‐term diseases, nature of laboratory tests), basic patient information (age, sex, birth date, death date, department and town of residence). It is linked to hospitalisation data (admission date and length of stay, principal and associated diagnoses using ICD10 codes, medical procedures and cost information on hospital stays). Data is available from 2006 for the main reimbursement program and the database has been progressively enriched with data from other programs, reaching quasi‐completeness in 2009. A medical causes of death registry was added in 2018, with satisfactory completeness since 2013. We only used de‐identified patient‐level data; therefore, individual informed consent was not required.

A major strength of the SNDS is its size and completeness of data for the French population. Patients are included from birth (or arrival in France) to death (or relocation). Main limitations of this database include the lack of primary care records, laboratory test results, and biological measurements.

The number of published studies using the SNDS has been increasing since its creation in 2016, with 79 published works in 2023 according to PubMed. Anyone (individual, hospital, charity, company) can access the database to conduct a study with a public interest. The SNDS is particularly used in healthcare provision studies, for the evaluation of healthcare policies, as well as for pharmacovigilance, drug and vaccine effectiveness studies, and health economics research [1].

From an earlier literature review [7], the SNDS has been used for signal detection in one project [20], but it was not part of the bigger multi‐database initiatives, particularly the OMOP initiative [12].

### 2.2 Study Design

The SCCS is a case‐only design comparing the event rate during exposed and unexposed time within the same individual [21]. Since all comparisons are made within person, time invariant, between person confounding is inherently addressed. It relies on a range of assumptions, detailed in the Supplement.

One limitation with this method is that time‐varying confounding is not handled by design. Active comparators have been recently implemented with SCCS [15] to reduce time‐varying confounding by indication. We therefore hypothesised that it might improve the signal detection performance of SCCS further and, as such, have been implemented in this study.

### 2.3 Study Population and Design Choices

Details on choosing the study design can be found in the Supplement, as this study builds on a common protocol applied in three different data sources, including the Clinical Research Practice Datalink (CPRD) and Merative MarketScan research databases. Some adjustments were needed to fit the structure of the SNDS. Specifically, only the later observation period was implemented due to data availability, and the second active comparator, cefalexin, was not used because of a lack of prescriptions in France. Drugs are coded based on the CIP13 system, which is specific to the SNDS. Diagnoses are coded based on the ICD‐10 classification, with the same codelists used in MarketScan.

The observation period lasted 2 years from the 1 January 2017 to the 31 December 2018. The risk window lasted 30 days and began on the day after the dispensing date. We conducted a crude primary analysis, assuming no relevant time‐varying confounders. The population consisted of all adults with at least one oral macrolide (azithromycin, erythromycin, clarithromycin) or fluoroquinolone (FQ) (ciprofloxacin, ofloxacin, levofloxacin, moxifloxacin) prescription during the observation period. Participants were eligible for inclusion at the latest of 1 year post‐registration or their 18th birthday. Amoxicillin was chosen as an active comparator as it has similar indications to macrolides and fluoroquinolones. More details can be found in the Supplement.

### 2.4 Reference Set

We built a reference set specifically for this study using a taxonomy framework to ensure the selected drug outcome pairs are well suited to the SCCS design. The full process has been detailed in a previous paper [22]. The reference set contains 104 positive controls (drug‐outcome pairs) and 58 negative controls in total, based on 30 outcomes from all organ classes.

Positive and negative control outcomes were selected among outcomes present on United Kingdom labels, on an individual drug basis, for consistency with other studies we are conducting. United Kingdom labels are almost identical to the French versions regarding adverse reactions. Only clinically important outcomes meeting the SCCS requirements and a priori appropriately captured in all databases of the project and therefore in the SNDS were considered in the reference set, based on a classification included in the taxonomy framework. More details can be found in the Supplement.

### 2.5 Statistical Analyses and Measures of Performance

We used conditional Poisson regression to estimate incidence rate ratios (IRRs) and incorporated active comparators using both simple ratio and effect modifier models. The ratio method is the simplest approach to estimate the active comparator rate ratio. The effect for each drug is estimated independently in two separate case series. The estimate is then calculated as the rate ratio for the risk period following the exposure to the DOI divided by the rate ratio for the risk period following an exposure to the comparator drug. Schultze et al. have provided details in a recent publication on why this estimand corresponds to the active comparator rate ratio [17]. The alternative approach for incorporating the active comparator consists of fitting a regression model with nested exposure variables. A new variable is introduced, corresponding to the risk period following an exposure to either the DOI or the comparator. The estimand is the active comparator RR, corresponding to the interaction term in the regression. Details on implementation can be found in [17]. For all models, the 95% confidence intervals (CIs) were calculated as 95% CI = exponential of (ln(IRR) ± 1.96*total standard error).

A positive signal was triggered when the lower bound of the IRR 95% CI was ≥ 1. Performance was measured using sensitivity and specificity with respect to the UK product labels and computing the resulting area under the curve (AUC) of the receiver operating characteristics (ROC).

Insufficient statistical power is a common reason for the inability to highlight safety signals, as outcomes are often rare. We therefore also examined the performance of the drug‐outcome pairs where there was apparent sufficient power. This also allows us to focus on drug‐event pairs where the ability of the active comparator to address potential indication bias has a greater chance of being highlighted. We considered satisfactory power for drug‐outcome pairs with at least five events in the risk window, and where the upper bound of the IRR 95% CI was < 2 effects with no evidence; for example, RR = 1.55 (95% CI: 0.90–2.10) would not be considered satisfactory power.

### 2.6 Sensitivity Analyses

As there is no end of registration in the data in the SNDS, except for death, we explored the impact of loss to follow‐up in the population. Follow‐up was terminated when there was a gap of > 6 months between two records of care utilisation.

## 3 Results

There were 754 350 patients who met the inclusion criteria. The mean length of follow‐up was 672.4 days (median: 729; SD: 144 days). The mean number of antibiotics dispensed during the observation period was 2.1 and varied slightly by drug. The mean age was 70.4 years in 2017 (median: 73; SD: 17.4). 17.3% of patients (130 492) died during the observation period. The main characteristics of the study population by drug class are presented in Table 1.

**TABLE 1:** Key characteristics of the study population by drug class.

	FQs	Macrolides	Amoxicillin
Number of patients	287 774	93 337	557 034
Age at cohort entry			
Mean (SD)	71.8 (15.9)	67.7 (17.9)	69.9 (17.8)
Median (IQR)	74 (21)	70 (25)	73 (25)
Sex (%)			
Female	45.7	53.7	50.9
Male	54.3	46.3	49.1
Mean length of follow‐up (days)	661.4	688.4	681.2
Number of antibiotic prescriptions*			
Mean (SD)	2.6 (2.3)	3.2 (3.3)	2.2 (1.9)
Median (IQR)	2 (2)	2 (3)	2 (2)
Proportion of people dying during the observation period (%)	21.3	13.1	15.1

* Any antibiotic considered in this study.

### 3.1 Main Analysis

The overall sensitivity and specificity of the SCCS without active comparator were 0.71 and 0.59, respectively. When restricting the reference set to drug‐outcome pairs with sufficient power, the sensitivity was 0.89, and specificity was 0.43. When using amoxicillin as an active comparator, the sensitivity decreased to 0.52, but the specificity increased to 0.91 (Table 2). Results using the nested model were similar to those with the simple ratio model (Supporting Information). Performance measures varied by drug class (Table 3).

**TABLE 2:** Measures of performance for the SCCS in the SNDS—simple ratio model.

	All pairs with at least one event during the risk or baseline period^a^		Pairs with enough power	
	No comparator	Amoxicillin	No comparator	Amoxicillin
Number of pairs	156	156	119	119
Sensitivity	0.71	0.41	0.89	0.52
Specificity	0.59	0.93	0.43	0.91
PPV	0.75	0.91	0.75	0.91
NPV	0.53	0.48	0.65	0.50
AUC	0.65	0.67	0.66	0.72

^a^ Six drug outcome pairs not looked at because of no events.

**TABLE 3:** Measures of performance for the SCCS in the SNDS by drug class—drug‐outcome pairs with sufficient power only.

Comparator	FQs		Macrolides	
	No comparator	Amoxicillin	No comparator	Amoxicillin
Sensitivity	0.96	0.66	0.73	0.18
Specificity	0.14	0.64	0.59	0.97
PPV	0.82	0.88	0.59	0.80
NPV	0.50	0.32	0.73	0.62
AUC	0.55	0.65	0.66	0.58

### 3.2 Sensitivity Analyses

Sixty five thousand seven hundred and seventy seven patients (8.7%) of the population were censored because they had a gap of > 6 months between two care utilisation episodes. The mean length of follow‐up was reduced by 10 days on average (mean length of follow‐up = 662.3 days). However, this did not considerably impact the method's performance: without active comparators, the sensitivity was 0.83 for pairs with enough power, and the specificity 0.49. Using amoxicillin as the active comparator, the sensitivity was 0.50 and the specificity 0.87, using sufficiently powered drug‐outcome pairs.

## 4 Discussion

The overall performance of SCCS in the SNDS for drug‐outcome pairs with enough power showed a sensitivity of 0.89 and specificity of 0.43. When using amoxicillin as an active comparator, the sensitivity decreased to 0.52, but the specificity increased to 0.91. Power was satisfactory for a majority of drug‐outcome pairs, except with moxifloxacin and erythromycin, drugs for which about half of the outcomes were insufficiently powered.

There is no standard definition of what sufficiently good performance is for signal detection, and this depends on how a method is implemented as part of an overall process [23]. In this study, the SCCS performed better than random chance (AUC of 0.5), with an AUC of 0.66. Although the specificity was lower than expected for potential reasons explored below, the SCCS identified a vast majority of positive controls in the SNDS and seems to be a potentially useful method for signal detection using RWD.

### 4.1 Use of Active Comparators

Using an active comparator led to a large increase in the specificity of the results, which suggests that this approach handled confounding by indication as expected and also increased overall performance in the SNDS (AUC of 0.72 with active comparators versus 0.66 without). However, sensitivity was greatly reduced. This is because all outcomes had an elevated RR with amoxicillin, which was partly unexpected as they were selected to be negative controls (absent from the comparator label in the United Kingdom). Possible explanations include confounding by indication, but also uncertainty regarding ‘gold standard’ reference sets in signal detection research: for example, vertigo and renal problems were labelled as adverse events in the French but not in the United Kingdom amoxicillin label [24, 25]. Individual regulatory authorities have varying perspectives on the evidential basis for potential adverse reactions, and labels are therefore specific to countries or regions, with occasional differences between them. Moreover, amoxicillin is the most prescribed antibiotic in France [26], and power was very large so even small increased risks had statistical strong evidence.

In signal detection, sensitivity is often favoured over specificity as the aim is to detect new ADRs [27]. Further hypothesis testing studies are then conducted to confirm or refute alerts highlighted in signal detection. However, in this study, the specificity was notably low (below 0.50) without an active comparator. In this case, using amoxicillin as an active comparator could be useful to achieve high levels of specificity, at the cost of missing some true positives. When deciding whether to apply active comparators, one should be aware of the trade‐off between sensitivity and specificity, and determine a level of tolerance for false positives versus missed signals. There are other challenges associated with the use of an active comparator for signal detection. It may be hard to choose an appropriate comparator a priori or to fully understand the anticipated safety profiles of appropriate comparators, particularly for signal detection of newer therapies where safety profiles are emerging and comparators may not exist. Exposed populations could also differ between the investigated products and the active comparator; for example, some antibiotics may be prescribed as second‐line treatment and to patients with different comorbidities compared to the comparator drug.

The impact of using active comparators varied widely by drug class. For fluoroquinolones, it enabled a substantial increase in specificity of up to 67% with a moderate loss of sensitivity < 30%. The general performance of the method in terms of the AUC was therefore improved. On the contrary, with macrolides, the loss in sensitivity was greater than the gain in specificity, and active comparators were less useful, especially in a signal detection context. This may be partly explained by the difference between the number of positive and negative controls in the two drug classes. Identifying a single comparator suitable for different drug classes is difficult, which may limit the usefulness of active comparators for signal detection if drugs with a wide range of indications are being investigated.

### 4.2 Reasons for False Positives

In our reference set, we deliberately included negative control outcomes prone to confounding by indication (e.g., pneumonia, cellulitis) to test the ability of active comparators to handle these. These infection‐related outcomes adversely affected the specificity of the method without an active comparator, since they led to significant RR, likely due to confounding by indication. In a real‐life study, careful consideration of the outcomes under study is needed to minimise potential confounding by indication, particularly if active comparators are not used.

Clarithromycin accounted for most of the false positives compared with other drugs in the main analysis (8 out of 23 false positives). While the elevated RR with upper gastro‐intestinal (GI) bleeding can be explained due to indications for treating bacterial duodenal ulcers, those for amnesia, gait disturbance, hip fracture, peripheral neuropathy, vasculitis, pancytopenia, and vision loss are more surprising, especially since they were not identified as false positives for other drugs of the same class. There is no evidence in the literature for any of these associations, except in the case of drug–drug interactions; for example, the interaction of clarithromycin with colchicine has been reported as causing fatal pancytopenia [28]. The false positive signal with hip fracture might be explained by a frequent co‐prescription of clarithromycin with omeprazole to treat ulcers, which can increase the risk of hip fracture [29]. There were some case reports of leukocytoclastic vasculitis [30] following clarithromycin treatment, but this is not mentioned in the label. Using amoxicillin as an active comparator, only the signal with anaemia remained, indicating that some of these false positives could be due to confounding by indication.

### 4.3 Comparison With Other Studies

Thurin et al. explored the SCCS for drug safety signal detection in the SNDS, using an outcome‐based approach. They investigated two outcomes, one of which was included in our reference set (upper GI bleeding). They explored a variety of drugs, but fluoroquinolones and macrolides were not included in their study for upper GI bleeding. Thurin et al. examined 96 different settings related to implementations of SCCS; the most discriminant variant (first outcome occurrence, risk window = period of dispensation) in their study had a 0.89 sensitivity and 0.57 specificity for acute liver injury, and a 0.74 sensitivity and 0.86 specificity for upper GI bleeding [3, 4]. Our study found AUCs in the same range as in this work, but lower than their most discriminant variant. However, we considered a different approach (drug‐based versus outcome‐based), a different reference set, and therefore potentially different imbalances between positive and negative controls in terms of raw counts and relative sample sizes. Our criteria for sufficiently powered drug‐outcome pairs were also different.

### 4.4 Strengths and Limitations

This study is one of the first large‐scale signal detection studies conducted in the SNDS, with 30 outcomes and seven drugs included in the reference set. All drugs and outcomes were theoretically well‐suited to the SCCS design, which has not been the case in previous method performance evaluation studies. These considerations of tailoring the study design to the drug–outcome pairs of interest should be applied to other signal detection studies, at least for performance assessment but also in real‐life studies, to avoid spurious or missed alerts from inappropriate outcomes. Although the evaluation was limited to antibiotics, the results shown here should generalise to any drug well‐suited to the SCCS requirements.

The SNDS data only contain hospital diagnoses and drugs delivered in community pharmacies. Therefore, there is an underestimation of the number of events, especially for the less severe outcomes. This applies both to the baseline and risk periods and is expected to impact on power rather than validity. In this study, the sample size was adequate for most of the reference set. Since antibiotics delivered in hospitals are not available in the database, there is potential misclassification of the exposure. However, hospital antibiotic consumption only represents 7% of all antibiotic consumption in France [31]. The SNDS therefore captures a majority of antibiotic prescriptions in France.

There is no perfect reference set for signal detection. Notably, labels do not necessarily represent true causal associations, and an outcome absent from the label could still be causally associated with the drug of interest if the association is unknown. Conversely, well‐known ADRs can be difficult to detect in observational data because the risks can be mitigated in medical practice [32]. In this study, there were also some minor differences between the UK labels that were used to build the reference set and the French labels.

Finally, we chose to apply the simplest form of SCCS design in this study. While more complex forms exist, for example accounting for time‐varying confounding or exposure‐event dependency, a routine signal detection application would generally not be well suited to the addition of such complexity. Future work could assess the implications of this approach.

## 5 Conclusions

Benefiting from its size, completeness of data, and length of follow‐up, the SNDS is a powerful data source for signal detection activities, particularly for outcomes captured in hospital. Using a carefully designed reference set of drug‐outcome pairs well suited to the study design, the SCCS showed satisfactory performance for signal detection. In this study using the SNDS, active comparators improved overall performance at the expense of greatly reduced sensitivity. Since sensitivity is generally preferred over specificity in signal generation, the utility of active comparators in this context should be determined on a case‐by‐case basis.

### 5.1 Plain Language Summary

Signal detection is the process of identifying possible new or unexpected side effects of a medicine by looking for patterns in routinely collected healthcare data. We explored the performance of a self‐controlled method, which is a method where risks of outcomes (potential adverse events) are compared between different time points in each patient, for the detection of Adverse Drug Reactions using a French administrative health claims database. We also explored the added value of a recent methodological development, active comparators, which are drugs used to treat similar health conditions but not reported to have adverse events of interest and can address some of the remaining bias. We looked at the ability of the method to identify known drug side effects for commonly prescribed antibiotics. The method was able to give a correct result for 89% (known adverse drug events) of positive controls and 43% of negative controls (drugs known not to be associated with certain outcomes), when limited to drug‐outcome pairs with a satisfying number of occurrences. The proportion of negative controls correctly identified increased to 91% when using active comparators, but that of positive controls decreased to 52%. Overall, the performance of the method was satisfactory in this study. The use of active comparators improved overall performance, at the cost of identifying fewer positive controls.

## Ethics Statement

As we used de‐identified patient‐level data, individual informed consent was not required. The study was approved by the London School of Hygiene and Tropical Medicine Research Ethics Committee (Reference: 27650). The French study protocol was approved by the National Data Protection Agency, CNIL.

## Conflicts of Interest

A.C. is funded by a GSK PhD studentship to undertake this work. A.B. is an employee of GSK and holds stocks and stock options. F.H. is an employee of GSK and holds financial equities in GSK. I.J.D. holds grants and shares from GSK. GSK markets the following drugs: Augmentin. However, A.B. and F.H. did not actively participate in the assessment of the labels and choice of outcomes for this methodological study.

## Supporting information




**Data S1:** Supporting Information.

## References

[1] Assurance Maladie , “Présentation du système national des données de santé (SNDS),” 2024.

[2] O. Maillard , R. Bun , M. Laanani , et al., “Use of the French National Health Data System (SNDS) in Pharmacoepidemiology: A Systematic Review in Its Maturation Phase,” Thérapie 79 (2024): 659–669. 38834394 10.1016/j.therap.2024.05.003

[3] N. H. Thurin , R. Lassalle , M. Schuemie , et al., “Empirical Assessment of Case‐Based Methods for Identification of Drugs Associated With Upper Gastrointestinal Bleeding in the French National Healthcare System Database (SNDS),” Pharmacoepidemiology and Drug Safety 29 (2020): 890–903. 32524701 10.1002/pds.5038

[4] N. H. Thurin , R. Lassalle , M. Schuemie , et al., “Empirical Assessment of Case‐Based Methods for Identification of Drugs Associated With Acute Liver Injury in the French National Healthcare System Database (SNDS),” Pharmacoepidemiology and Drug Safety 30 (2021): 320–333. 33099844 10.1002/pds.5161

[5] S. Gruber , A. Chakravarty , S. R. Heckbert , et al., “Design and Analysis Choices for Safety Surveillance Evaluations Need to Be Tuned to the Specifics of the Hypothesized Drug–Outcome Association,” Pharmacoepidemiology and Drug Safety 25 (2016): 973–981. 27418432 10.1002/pds.4065

[6] M. Arnaud , B. Bégaud , N. Thurin , N. Moore , A. Pariente , and F. Salvo , “Methods for Safety Signal Detection in Healthcare Databases: A Literature Review,” Expert Opinion on Drug Safety 16, no. 6 (2017): 721–732. 28490262 10.1080/14740338.2017.1325463

[7] A. Coste , A. Wong , M. Bokern , A. Bate , and I. J. Douglas , “Methods for Drug Safety Signal Detection Using Routinely Collected Observational Electronic Health Care Data: A Systematic Review,” Pharmacoepidemiology and Drug Safety 32, no. 1 (2022): 28–43. 36218170 10.1002/pds.5548 PMC10092128

[8] M. J. Schuemie , P. M. Coloma , H. Straatman , et al., “Using Electronic Health Care Records for Drug Safety Signal Detection: A Comparative Evaluation of Statistical Methods,” Medical Care 50 (2012): 890–897, http://ovidsp.ovid.com/ovidweb.cgi?T=JS&PAGE=reference&D=med9&NEWS=N&AN=22929992. 22929992 10.1097/MLR.0b013e31825f63bf

[9] S. N. Murphy , V. Castro , J. Colecchi , A. Dubey , V. Gainer , and C. Herrick , “Partners HealthCare OMOP Study Report,” 2011.

[10] P. B. Ryan , P. E. Stang , J. M. Overhage , et al., “A Comparison of the Empirical Performance of Methods for a Risk Identification System,” Drug Safety 36, no. S1 (2013): S143–S158, http://ovidsp.ovid.com/ovidweb.cgi?T=JS&PAGE=reference&D=med10&NEWS=N&AN=24166231. 24166231 10.1007/s40264-013-0108-9

[11] P. B. Ryan , D. Madigan , P. E. Stang , et al., “Empirical Assessment of Methods for Risk Identification in Healthcare Data: Results From the Experiments of the Observational Medical Outcomes Partnership,” Statistics in Medicine 31 (2012): 4401–4415, http://ovidsp.ovid.com/ovidweb.cgi?T=JS&PAGE=reference&D=med9&NEWS=N&AN=23015364. 23015364 10.1002/sim.5620

[12] M. J. Schuemie , R. Gini , P. M. Coloma , et al., “Replication of the OMOP Experiment in Europe: Evaluating Methods for Risk Identification in Electronic Health Record Databases,” Drug Safety 36 (2013): 159–169, http://ovidsp.ovid.com/ovidweb.cgi?T=JS&PAGE=reference&D=emed14&NEWS=N&AN=372188901. 10.1007/s40264-013-0109-8 24166232

[13] M. J. Schuemie , F. Arshad , N. Pratt , et al., “Vaccine Safety Surveillance Using Routinely Collected Healthcare Data—An Empirical Evaluation of Epidemiological Designs,” Frontiers in Pharmacology 13 (2022): 13. 10.3389/fphar.2022.893484 PMC9299244 35873596

[14] M. J. Schuemie , M. S. Cepede , M. A. Suchard , et al., “How Confident Are we About Observational Findings in Healthcare: A Benchmark Study,” Harvard Data Science Review 2, no. 1 (2020), 10.1162/99608f92.147cc28e. PMC7755157 33367288

[15] J. Hallas , H. Whitaker , J. A. Delaney , S. M. Cadarette , N. Pratt , and M. Maclure , “The Use of Active Comparators in Self‐Controlled Designs,” American Journal of Epidemiology 190 (2021): 2181–2187. 33861309 10.1093/aje/kwab110

[16] Y. Zhang , M. Fan , N. T. Y. Tsie , et al., “Association Between Oral Fluoroquinolones and Neuropsychiatric Events: Self‐Controlled Case Series With Active Comparator Design,” Pharmacoepidemiology and Drug Safety 33 (2024): e70036. 39420659 10.1002/pds.70036

[17] A. Schultze , J. Brown , J. Logie , et al., “Overcoming Time‐Varying Confounding in Self‐Controlled Case Series With Active Comparators: Application and Recommendations,” American Journal of Epidemiology 194, no. 1 (2024): 220–225, https://academic.oup.com/aje/advance‐article/doi/10.1093/aje/kwae216/7716740. 10.1093/aje/kwae216 PMC11735952 39030722

[18] L.‐M. Scailteux , C. Droitcourt , F. Balusson , et al., “French Administrative Health Care Database (SNDS): The Value of Its Enrichment,” Pharmacoepidemiology 74, no. 2 (2018): 215–223. 10.1016/j.therap.2018.09.072 30392702

[19] C. Collin , F. Morand , and F. Maurel , “Why France is the Go‐To Country to Conduct Real‐World Studies,” 2023.

[20] N. H. Thurin , R. Lassalle , M. Schuemie , et al., “Empirical Assessment of Case‐Based Methods for Drug Safety Alert Identification in the French National Healthcare System Database (SNDS): Methodology of the ALCAPONE Project,” Pharmacoepidemiology and Drug Safety 29 (2020): 993–1000, http://ovidsp.ovid.com/ovidweb.cgi?T=JS&PAGE=reference&D=mesx&NEWS=N&AN=32133717. 32133717 10.1002/pds.4983

[21] M. A. Suchard , I. Zorych , S. E. Simpson , D. Madigan , M. J. Schuemie , and P. B. Ryan , “Empirical Performance of the Self‐Controlled Case Series Design: Lessons for Developing a Risk Identification and Analysis System,” Drug Safety 36 (2013): S83–S93, http://ovidsp.ovid.com/ovidweb.cgi?T=JS&PAGE=reference&D=emed14&NEWS=N&AN=372188895. 24166226 10.1007/s40264-013-0100-4

[22] A. Coste , A. Y. S. Wong , C. Warren‐Gash , J. Matthewman , A. Bate , and I. J. Douglas , “Implementation of a Taxonomy‐Based Framework for the Selection of Appropriate Drugs and Outcomes for Real‐World Data Signal Detection Studies,” Drug Safety 47 (2023): 183–192. 38093083 10.1007/s40264-023-01382-5 PMC10821990

[23] A. Bate and S. J. W. Evans , “Quantitative Signal Detection Using Spontaneous ADR Reporting,” Pharmacoepidemiology and Drug Safety 18 (2009): 427–436. 19358225 10.1002/pds.1742

[24] Electronic Medicines Compendium , “Amoxicillin 500mg Capsules,” accessed September 28, 2025, https://www.medicines.org.uk/emc/product/526/smpc.

[25] “Base de données publique des médicaments,” AMOXICILLINE ARROW LAB 500 mg, gélule, accessed September 28, 2025, https://base‐donnees‐publique.medicaments.gouv.fr/medicament/66916798/extrait.

[26] Santé publique France , “Consommation d'antibiotiques en secteur de ville en France 2011–2021”.

[27] A. C. Păcurariu , “The Role of Signal Detection in Pharmacovigilance,” 2018.

[28] I. F. N. Hung , A. K. L. Wu , V. C. C. Cheng , et al., “Fatal Interaction Between Clarithromycin and Colchicine in Patients With Renal Insufficiency: A Retrospective Study,” Clinical Infectious Diseases 41, no. 3 (2005): 291–300, https://academic.oup.com/cid/article/41/3/291/336398. 16007523 10.1086/431592

[29] B. K. S. Thong , S. Ima‐Nirwana , and K. Y. Chin , “Proton Pump Inhibitors and Fracture Risk: A Review of Current Evidence and Mechanisms Involved,” International Journal of Environmental Research and Public Health 16, no. 9 (2019): 1571. 31060319 10.3390/ijerph16091571 PMC6540255

[30] S. R. Gavura and S. Nusinowitz , “Leukocytoclastic Vasculitis Associated With Clarithromycin,” Annals of Pharmacotherapy 32 (1998): 543–545. 9606474 10.1345/aph.17286

[31] K. Hider‐Mlynarz and C.‐O. Betansedi , “La consommation des antibiotiques en France de 2000 a 2020,” 2023.

[32] G. N. Norén , O. Caster , K. Juhlin , and M. Lindquist , “Zoo or Savannah? Choice of Training Ground for Evidence‐Based Pharmacovigilance,” Drug Safety 37 (2014): 655–659. 25005708 10.1007/s40264-014-0198-z

