# Supplementary material for: Performance of the Self‐Controlled Case Series With Active Comparators for Drug Safety Signal Detection Using the French Administrative Healthcare Database (SNDS)
Source: Pharmacoepidemiol Drug Saf. 2025 Nov 14;34(11):e70224. doi: 10.1002/pds.70224 (PMC12616763; doi:10.1002/pds.70224)
Supplement: Supplementary file 1 — Data S1: Supporting Information. [file PDS-34-e70224-s001.docx]

## Details on multiple prescriptions and overlaps

If there were two or more prescriptions of the same drug with a gap shorter than 30 days, they were considered as continuous exposure with an extension of the risk window ending 30 days after the start of the last exposure. If the gap was longer than 30 days, the exposures were treated separately (Figure 3). When there were two or more concomitant or overlapping exposures of different antibiotics of interest, the corresponding risk periods were excluded. A 30-day pre-exposure window was introduced before every exposure due to the assumption that the outcome does not affect future exposure [21]. In the case of an overlapping risk period and pre-exposure risk window, the overlapping period was considered as risk period and the pre-exposure period was shortened.


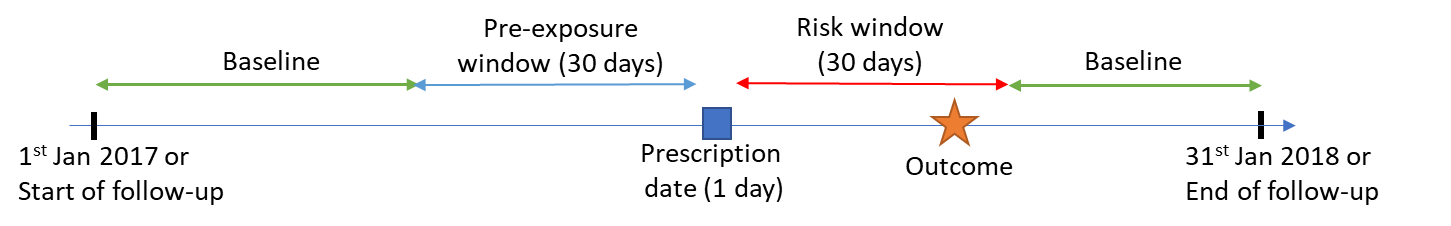


**Figure 1 – Summary of study design choices**


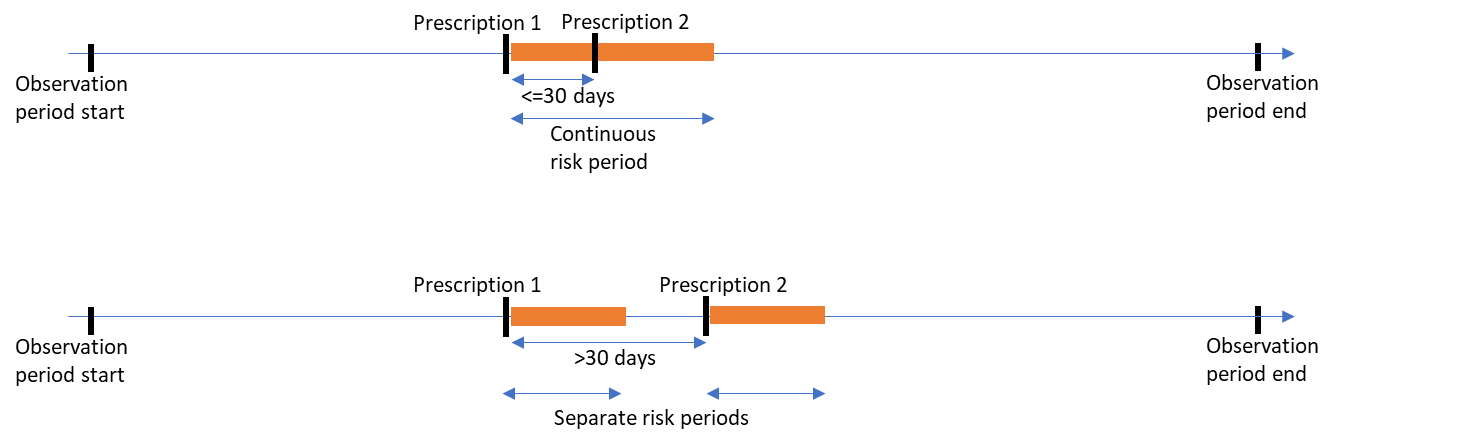
**Figure 2 – Summary of the risk periods for the study in the case of multiple prescriptions**

## Table 1 – Numbers of patients included in the study

|  | **Number of patients FQs** | **Number of patients macrolides** | **Number of patients amoxicillin** | **Number of patients cefalexin** |
| --- | --- | --- | --- | --- |
| **Incident 97/98** | 15,503 | 43,519 | 91,938 | 17,520 |
| **Incident 17/18** | 34,094 | 123,290 | 261,853 | 22,652 |
| **All events* 97/98** | 18,548 | 51,623 | 108,998 | 21,065 |
| **All events* 17/18** | 44,933 | 161,408 | 338,700 | 29,863 |

*All events with at least 12 months from the previous outcome occurrence

## Table 2 - Measures of performance for the SCCS in CPRD – 2017/2018 observation period – Nested model

|  | Pairs with enough power | |
| --- | --- | --- |
|  | **Amoxicillin** | **Cefalexin** |
| **Sensitivity** | 15.9 | 35.3 |
| **Specificity** | 92.3 | 95.7 |
| **PPV** | 77.8 | 92.3 |
| **NPV** | 39.3 | 50.0 |

## Sensitivity analysis: All events with a one-year outcome-free period

**Table 3 - Measures of performance for the SCCS in CPRD – 2017/2018 observation period – Sensitivity analysis including multiple outcomes per patient**

|  | All pairs with at least one event during the risk period | | | Pairs with enough power | | |
| --- | --- | --- | --- | --- | --- | --- |
|  | **No comparator** | **Amoxicillin** | **Cefalexin** | **No comparator** | **Amoxicillin** | **Cefalexin** |
| **Number of pairs** | 132 | 132 | 132 | 83 | 77 | 72 |
| **Sensitivity** | 0.30 | 0.13 | 0.19 | 0.50 | 0.22 | 0.35 |
| **Specificity** | 0.73 | 0.91 | 0.93 | 0.61 | 0.89 | 0.89 |
| **PPV** | 0.66 | 0.73 | 0.84 | 0.66 | 0.79 | 0.84 |
| **NPV** | 0.37 | 0.36 | 0.39 | 0.44 | 0.40 | 0.43 |
| **AUC** | 0.52 | 0.52 | 0.56 | 0.56 | 0.56 | 0.62 |
